# Radioprotection of thymine and calf thymus DNA by an azo compound: mechanism of action followed by DPPH radical quenching & ROS depletion in WI 38 lung fibroblast cells

**DOI:** 10.1016/j.heliyon.2020.e04036

**Published:** 2020-05-29

**Authors:** Durba Ganguly, Ramesh Chandra Santra, Swagata Mazumdar, Abhijit Saha, Parimal Karmakar, Saurabh Das

**Affiliations:** a Department of Chemistry (Inorganic Section), Jadavpur University, Kolkata 700032, India; b Department of Life Science and Biotechnology, Jadavpur University, Kolkata 700032, India; c UGC-DAE CSR, Kolkata Centre, Sector III, LB- 8, Bidhan Nagar, Kolkata 700 098, India

**Keywords:** Pharmaceutical chemistry, 2-(2-hydroxyphenylazo)-indole-3^∕^-acetic acid (HPIA), Thymine, Calf thymus DNA, DPPH, DCFDA, Radioprotection

## Abstract

**Purpose:**

To explain the observed radio-protection properties of an azo compound, 2-(2-hydroxyphenylazo)-indole-3^∕^-acetic acid (HPIA).

**Materials and methods:**

Mechanism of radioprotection by HPIA was attempted using the stable free radical 2, 2-diphenyl-1-picrylhydrazyl (DPPH) using UV-Vis and electron paramagnetic resonance (EPR) spectroscopy. The radical destroying ability of HPIA was studied by depletion of reactive oxygen species (ROS) in WI 38 lung fibroblast cells.

**Results & Discussion:**

Studies indicate HPIA interacts with radical intermediates formed in solution following irradiation by ^60^Co γ-rays. As a result, reactive radical intermediates do not cause any damage on chosen substrates like thymine or calf thymus DNA when irradiated in presence of HPIA. The study showed that reactive intermediates not only react with HPIA but that the kinetics of their reaction is definitely faster than their interaction either with thymine or with DNA. Had this not been the case, much more damage would have been observed on chosen substrates following irradiation with ^60^Co γ-rays, in the presence of HPIA than actually observed in experiments, particularly those that were performed in a relatively high dose. Experiments reveal radiation induced-damage caused to thymine in presence of HPIA was ~ $\frac{1}{36}$ to ~ $\frac{1}{32\;}\;$times that caused in its absence under different conditions indicating the radio-protection properties of HPIA. In case of calf thymus DNA, damage in presence of HPIA was much lower than in its absence. A fluorometric microplate assay for depletion of ROS by detecting the oxidation of 2′,7′-dichlorofluorescin-diacetate (DCF-DA) into the highly fluorescent compound 2′,7′ dichlorofluorescein (DCF) indicated HPIA brought about a considerable check on ROS-mediated damage to cells by scavenging them right away.

**Conclusion:**

The study indicates HPIA may be an antioxidant supplement during radiotherapy.

## Teaser

Pharmaceutical chemistry, 2-(2-hydroxyphenylazo)-indole-3ï,¤-acetic acid (HPIA), Thymine, Calf thymus DNA, DPPH, DCFDA, Radioprotection

## 1 Introduction

Radiotherapy is commonly employed as part of a management of a wide variety of malignancies. To achieve local control, either alone or in combination with other modalities chemotherapy and/or surgery is used. It is reported almost half of all cancer patients receive radiotherapy as part of their treatment [1]. Hence, irradiation of non-cancerous normal tissues during therapeutic radiation results in side effects that include self-limited acute toxicities, mild chronic symptoms, and aspects of organ dys-function [2]. Improvement of chemical modifiers that can control radiation induced injury to normal tissue is getting increased attention and becoming important for reducing toxicities associated with therapeutic radiation [3]. The mechanism by which damage to normal tissues is prevented is called radio-protection and the compounds involved are known as radio-protectors [4]. These are administered either prior to or shortly after radiation exposure to alter response of normal tissues towards radiation and is considered an essential component of radiotherapy [5].

Although several applications of azo compounds related to biological systems are reported [6, 7, 8, 9, 10, 11, 12] not many have investigated them for their radio-protective properties. Although there is a study showing radio-protection by an azo compound, details regarding the process were not shown [13]. The study was however interesting since not only did it show another application of an azo compound, it highlighted an useful biological attribute that might be exploited in future [13]. In this study aspects related to radio-protection were investigated using 2-(2-hydroxyphenylazo)-indole-3^∕^-acetic acid (HPIA), an azo compound, whose preparation and characterization was reported earlier [7]. Since not much is known on radio-protection by azo compounds, and because the compound we worked with (HPIA) was not much toxic to normal cells as realized from the IC_50_ values on HEK 293T cells in a previous study [7], unlike most reports on azo compounds [14], we performed a detailed study with it to identify the mechanism by which compounds with azo bonds might provide protection against toxic and injurious effects of ionizing radiation [15].

The major attribute that a compound must possess in order to provide protection against damage caused by ionizing radiation is an ability to quench free radicals generated as a consequence of the radiolysis of water [16, 17, 18]. It is now established that interaction of ionizing radiation e.g. γ rays with water forms radical and molecular products [H·, ·OH, e_aq_¯, H_2_, H_2_O_2_ and H_3_O^+^] [16, 17, 18, 19]. In N_2_O saturated medium, e_aq_¯ is converted to an equivalent amount of ·OH, according to Eq. (1) [20].

(1) e_aq_¯ + N_2_O →·OH + N_2_O + OH¯

The products interact with a biological target modifying it in a manner having severe consequences in biology. Obviously then, preventing this from happening becomes important particularly with regard to protecting normal cells during radiotherapy [2, 3, 4, 5]. Thymine and calf thymus DNA were used as biological targets in model studies where they were irradiated in the absence and presence of HPIA by ^60^Co γ-rays in de-aerated (Ar saturated) and N_2_O saturated medium. To establish the mechanism by which radio-protection by HPIA is possible, radical scavenging experiments were designed and performed in the presence of the stable radical DPPH [21, 22, 23]. To further establish if radio-protection of a biological target was due to scavenging of free radicals within cells, 2‛,7‛-dichlorofluorescin diacetate (DCF-DA) ROS depletion assay was performed on WI-38 lung fibroblast cells [24, 25, 26, 27, 28]. It is worth mentioning here that we have been exploring biological attributes of complex formation of azo compounds for quite some time now and most of that work has dealt with the aspect of modifying an azo compound through complex formation leading to decreased formation of substances responsible for toxicity of azo compounds [7, 9, 10, 11]. Some of these studies also show that such modified azo compounds (complexed to metal ions) were more efficient in killing cancer cells than normal cells. This study shows azo compounds can be useful in protecting normal cells during radiotherapy as well.

## 2 Experimental

### 2.1 Materials

2-amino phenol and indole-3-acetic acid (IAA) [E. Merck, India] were used for preparing 2-(2-hydroxyphenylazo)-indole-3^∕^-acetic acid (HPIA) by diazo-coupling and re-crystallized from an ethanol-water mixture [7]. Sodium nitrate of analytical grade (E. Merck, India) was used to maintain the ionic strength of the medium. Stock solutions of HPIA were either prepared in ethanol or in DMSO. All solvents were procured either from E. Merck, India or E. Merck, Germany. Thymine (CAS No. 65-71-4) and calf thymus DNA (CAS No. 73049-39-5) were purchased from Sisco Research Laboratories (SRL), India. Sodium dihydrogen phosphate and disodium hydrogen phosphate [E. Merck, India] were used to prepare phosphate buffer solutions. Calf thymus DNA was dissolved in triple distilled water containing NaCl, KCl and MgCl_2_ (E. Merck, India). Absorbance of DNA solutions was noted at 260 and 280 nm respectively to calculate A_260_/A_280_. Ratio of absorbance at 260 and 280 nm provides an estimate of the purity of DNA. The ratio (1.8 < A_260_/A_280_ > 1.9) obtained indicates DNA was sufficiently free of protein. Concentration was measured in terms of nucleotide taking molar extinction coefficient at 260 nm to be 6,600 M^−1^cm^−1^. Ethidium bromide (EB) (CAS No. 1239-45-8) purchased from SRL, India was used as the fluorescent probe to determine the extent of damage caused to double stranded calf thymus DNA. DPPH used for radical scavenging studies was purchased from SRL, India. Pure N_2_O and Ar gases used for purging experimental solutions prior to irradiation with ^60^Co γ-rays were purchased from Indian Refrigeration Stores, Kolkata, India.

### 2.2 Equipments

Absorption spectra were recorded on JASCO V-630 Spectrophotometer, Japan. Fluorescence was measured on JOBIN YVON Fluoromax spectrophotometer. A pair of 10 × 10 mm quartz cuvette was used for absorption and fluorescence experiments. A pH meter [Equiptronix, EQ-610] was used for recording pH. During radiation chemical experiments, solutions were subjected to γ-irradiation from a ^60^Co source having dose rate of 3.1 KGy/min (monitored by Fricke dosimetry). Loss of thymine was determined by HPLC (Shimadzu Corporation, Japan). A C_18_ column (Phenomenex) was used as the stationary phase. Change was followed at 254 nm using 5% methanol and 95% water as mobile phase. Flow rate was 1 ml per minute. DPPH radical scavenging study was done with the help of UV-Vis and EPR spectroscopy. EPR measurements were made on Jeol JES-FA 200 ESR spectrometer equipped with a Jeol microwave bridge. Spectroscopic parameters were 9.44 GHz (frequency), 100 mT (field sweep), 0.998 mW (microwave power) and modulation amplitude 3000 mT. EPR of samples were recorded in Jeol Quartz pyrex EPR tube no. 193 5D.

### 2.3 Methods

#### 2.3.1 Radiation chemical experiments

Aqueous solutions of thymine with or without HPIA were prepared. Concentration of thymine in an experimental solution was 10^−4^ M while that of HPIA was 10^−5^M. Prior to irradiation aqueous solutions of samples were saturated with Ar or N_2_O by purging them with the gases for at least 30 min. Following irradiation of thymine at different dose in the absence or presence of HPIA, solutions were analyzed by HPLC. Concentration of radiolysed thymine was calculated from the peak area of the HPLC chromatograms.

Calf thymus DNA (50 μM), in phosphate buffer, was irradiated with ^60^Co γ-rays at different dose (8 Gy, 12 Gy, 16 Gy, 20 Gy, 24 Gy). EB was added to aliquots of such irradiated DNA samples and fluorescence was recorded in the range 540 nm–640 nm following an excitation at 510 nm. Emission maxima of the EB-DNA adduct was obtained at 590–600 nm and served as a measure of the DNA remaining intact [29, 30, 31]. Since reports suggest fluorescence intensity of the EB-DNA adduct is dependent on the concentration of EB, hence for maximum binding/saturation, concentration of EB was maintained at 800 μM (~16 folds higher than the concentration of DNA in the experiment).

#### 2.3.2 Radical scavenging experiments

##### 2.3.2.1 DPPH radical scavenging activity by electronic absorption spectroscopy

DPPH radical scavenging activity of HPIA was measured using UV-Vis spectroscopy. Owing to the presence of an odd electron, DPPH shows a strong absorption band at 517 nm. This gradually decreases if the odd electron pairs up in the presence of a radical scavenger, which was followed during the course of the experiment [21, 22, 23]. There are not many studies that indicate interaction of an azo compound with DPPH, not to mention any that aims to establish an application where an azo compound by virtue of its attribute of quenching DPPH might become biologically useful [32]. For our experiments, strength of the stock solution of DPPH being 6 mM, amount of DPPH taken for each experiment was 10 μL to arrive at a final concentration of 60 μM. The total volume of an experimental solution was 1mL (1000 μL). A reduced volume cuvette was used for the purpose. HPIA was taken from a stock of 3 mM in ethanol and appropriate amounts were added to each experimental solution to obtain final concentrations in the range 0–30 μM.

##### 2.3.2.2 DPPH radical scavenging by EPR spectroscopy

Electron paramagnetic resonance (EPR) measurements were performed at room temperature (298 K) to check the stability of a freshly prepared solution of DPPH in methanol [33, 34, 35, 36] for 30 min. No significant loss in signal was detected. To a 6 mM DPPH solution, different concentrations (0–5 mM) of HPIA were added. Solutions were mixed thoroughly and EPR signals were recorded 2 min after mixing HPIA and DPPH under identical conditions. DPPH denotes the radical DPPH•.

##### 2.3.2.3 Estimation of ROS by the DCFDA assay

The cell permeant reagent DCF-DA, a fluorogenic dye measures the activity of hydroxyl, peroxyl and other reactive oxygen species (ROS) within a cell [24, 25, 26, 27, 28]. After diffusion in to the cell, DCF-DA gets de-acetylated by cellular esterases to a non-fluorescent form, later oxidized by ROS to 2‛,7‛–dichlorofluorescein (DCF), another highly fluorescent compound showing green fluorescence. Fluorescence due to DCF was detected using a fluorescence spectrophotometer (Hitachi, Japan). Excitation was done at 504 nm and emission measured at 529 nm. Stock solutions of DCF-DA (10 mM) were prepared in methanol and diluted further with culture medium to 100 μM. Cells were then treated with different concentrations of HPIA (0 μM, 1 μM, 5 μM, 10 μM) and allowed to stand for 30 min. ROS was induced by irradiation of cells with the help of ^60^Co γ rays. Cells were washed with ice cold Hanks balanced salt solution (HBSS) and incubated with 100 μM DCF-DA for 30 min at 37 °C. Subsequently, cells were lysed with alkali and fluorescence was recorded.

## 3 Results and discussion

### 3.1 Radiation chemical experiments on thymine

2 × 10^−4^ M thymine was used in experiments to enable the application of high dose. This was necessary to ascertain the changes caused on thymine accurately when its aqueous solution was irradiated with ^60^Co γ rays. Since high concentrations were used, response obtained for a damage of the target can be said to be devoid of error that might occur for low concentrations.

**Figure 1:**
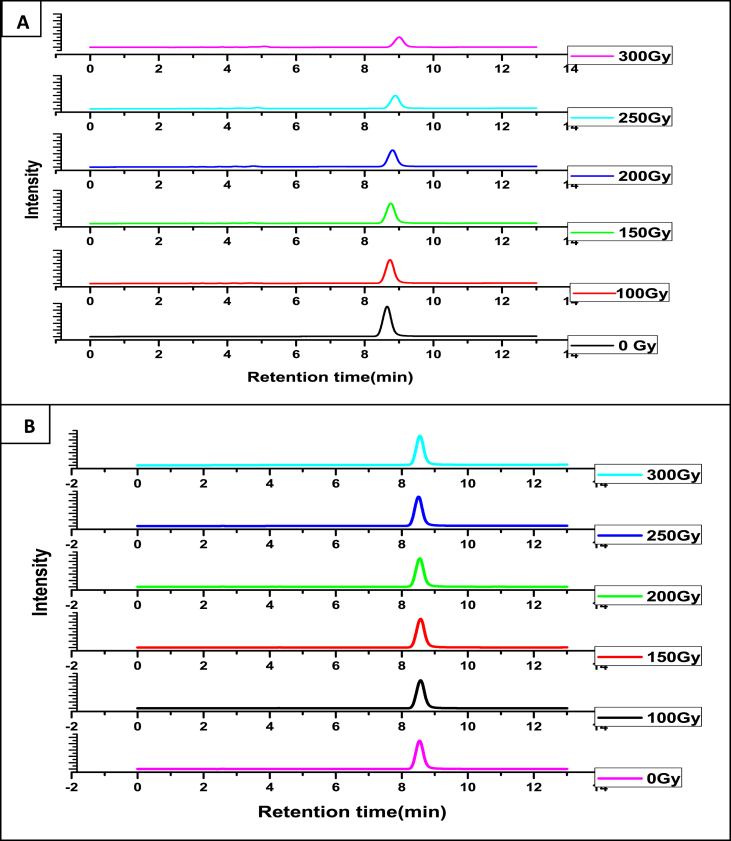
HPLC chromatograms recorded at 254 nm when 1 × 10^−4^ mol dm^−3^ thymine solution was irradiated with ^60^Co γ rays at different dose in absence (A) and presence of 1 × 10^−5^ mol dm^−3^ HPIA (B) under Ar saturated conditions.

**Figure 2:**
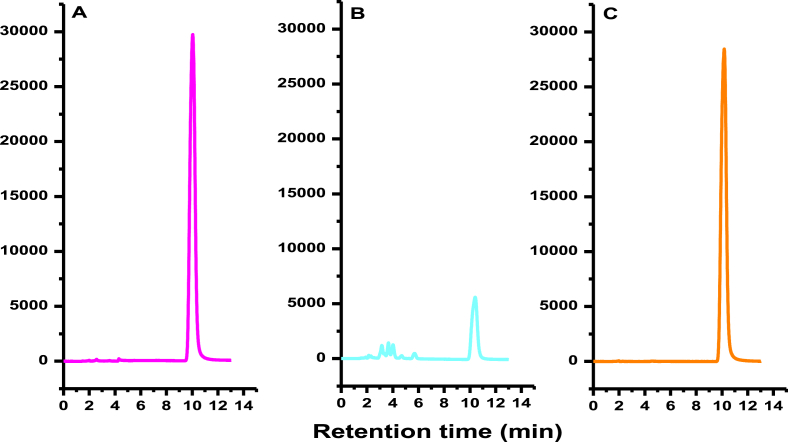
HPLC chromatograms of 1 × 10^−4^ mol dm^−3^ thymine recorded at 254 nm when subjected to (A) no radiation; (B) radiation of 200 Gy in absence of HPIA and (C) radiation of 200 Gy in presence of 1 × 10^−5^ mol dm^−3^ HPIA. Radiation provided under N_2_O saturated conditions.

From the extent of damage caused to thymine under Ar and N_2_O saturated conditions it was realized that HPIA provides significant protection to thymine compared to situations when it was subjected to irradiation in absence of HPIA. In fact, radiation-induced damage of thymine decreased enormously showing almost no change in presence of HPIA both in Ar and N_2_O saturated conditions. Figure 1A is the HPLC profile for the degradation of thymine with dose in the absence of HPIA in an Ar saturated medium while Figure 1B was obtained in presence of HPIA under exactly identical conditions. Degradation of thymine due to γ rays was also followed under N_2_O saturated conditions at different dose. Figure 2 is a representative HPLC profile for thymine under different conditions and helps us to realize that HPIA provides significant protection to thymine. HPLC profiles of Figure 2A (in absence of radiation) & Figure 2C (with radiation under similar conditions but in presence of HPIA) are similar while that of Figure 2B (with radiation under similar conditions but in absence of HPIA) is different.

**Figure 3:**
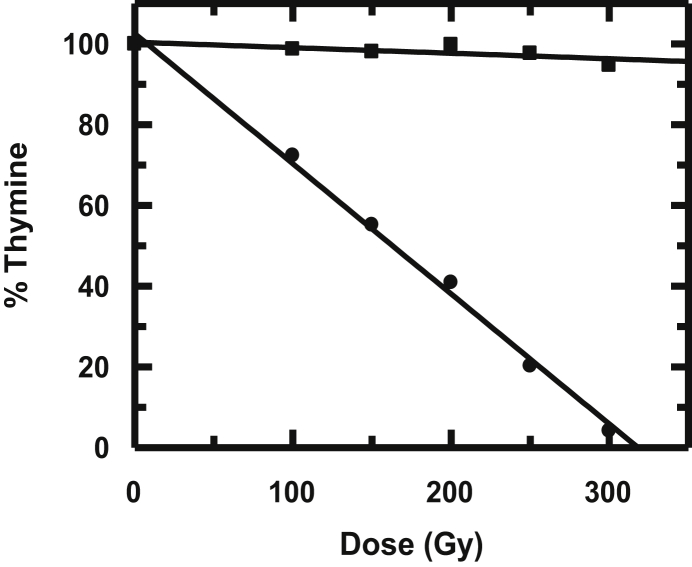
Amount of thymine remaining when subjected to γ-irradiation in the absence (●) and presence of HPIA (■) under N_2_O saturated conditions.

G (-thymine) was calculated for Ar and N_2_O saturated conditions (Table 1). Extent of degradation of thymine following irradiation in the absence and presence of HPIA in a N_2_O saturated medium was analyzed using HPLC (Figure 3). Table 1 clearly indicates HPIA is a radio-protector since it prevents degradation of thymine due to γ irradiation.

**Table 1:** G (-thymine) under different experimental conditions.

Additive	G (-thymine)	
	Ar saturated	N_2_O saturated
―	2.17	3.87
HPIA	0.06	0.12

Many possibilities exist how this might be possible. HPIA could be interacting with the products of the radiolysis of water (e^-^_aq_, ·OH and ·H) for an Ar saturated medium or with ·OH for an N_2_O saturated medium, thus preventing them from interacting with thymine. It could interact with the radical products formed on thymine, following an initial attack on it by the products of the radiolysis of water and then reverse changes that might have occurred. It could be a combination of both these processes. Hence, to identify the mechanism by which HPIA prevents radiation-induced damage of thymine we decided to perform experiments with DPPH making use of a scavenging assay [33, 34, 35, 36, 37].

### 3.2 Radiation chemical experiments on calf thymus DNA

**Figure 4:**
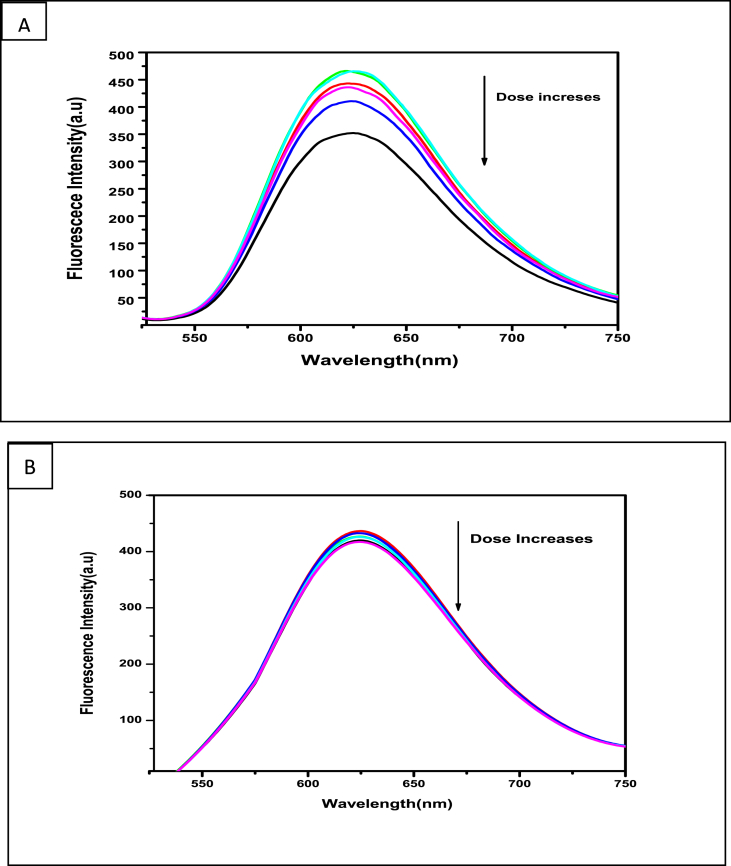
Fluorescence spectra of calf thymus DNA saturated with EB following irradiation by ^60^Co γ rays at different dose in the absence (A) and presence (B) of HPIA. [Calf thymus DNA] = 50 μM, [HPIA] = 10 μM, [EB] = 800 μM.

To realize the consequences of interaction of γ-radiation with DNA in the absence and presence of HPIA, experiments were performed in N_2_O saturated medium where formation of ·OH is maximum [20]. It is well established ·OH is the main species responsible for modification of double stranded DNA, hence experiments related to DNA were performed only in N_2_O saturated medium [38, 39, 40]. Figure 4A, shows a gradual decrease in fluorescence of calf thymus DNA treated with EB following irradiation with ^60^Co γ rays in the absence of HPIA. However, when DNA from the same stock was irradiated at different dose in presence of HPIA there was hardly any decrease in fluorescence following treatment with EB (Figure 4B) clearly indicating HPIA provides protection to DNA.

**Figure 5:**
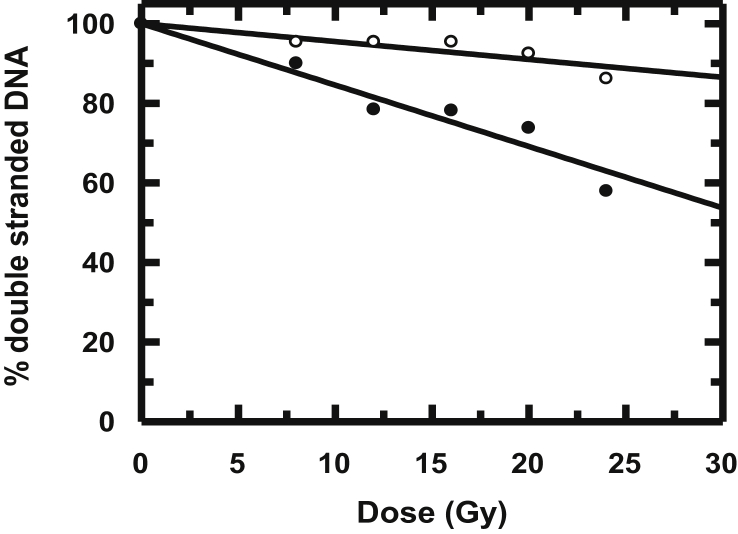
Amount of double stranded calf thymus DNA remaining following irradiation of a 50 μM solution in the absence (●) and presence of HPIA = 10 μM (o).

Figure 5 shows the dose-effect curves for the modification of double-stranded calf thymus DNA irradiated in the absence and presence of HPIA. Plots were exponential with dose. D_37_ was calculated from initial slopes of each plot. Percentage loss of double-stranded DNA due to irradiation was calculated using [DNA]_0_/D_37_. [DNA]_0_ indicates initial concentration of calf thymus DNA used for irradiation. The value [DNA]_0_/D_37_ is independent of DNA concentration in the base pair region in which our investigations were performed. Following irradiation in the presence of HPIA, double stranded DNA remained almost unaffected as realized from the dose-effect curve in Figure 5. Fluorescence intensity of DNA bound EB (Figure 4B) for all radiation dose, not only remained constant with respect to the control, rather at each individual dose fluorescence intensity of the DNA-EB adduct was higher than when DNA was irradiated in the absence of HPIA, indicating that at all individual irradiation, HPIA provides almost complete protection to DNA against radiation-induced damage. If this wasn't the case, at least at higher dose there should have been greater decrease in fluorescence of the DNA-EB adducts.

Interaction of radiation with water produces reactive free radicals [·OH, ·H., e_aq_^−^, H_2_O_2_] that are toxic if allowed to interact with several macromolecules in a biological system. These radicals affect the three dimensional structure of bio-molecules preventing them from performing their assigned functions. Hence, irradiation that is not under control initiates the breakdown of several important bio-molecules, a phenomenon commonly referred to as adverse effects of ionizing radiation. Using HPIA, this study made an effort to quench free radicals produced by irradiation, before they could cause undesired changes on a biological target, which extrapolated, might help to decrease toxic side effects associated with any ionizing radiation during radiotherapy,. There are studies on flavonoids procured from different sources and on tea extracts (catechins) etc. in this regard but not with an azo compound [39, 40, 41, 42]. Since azo derivatives are already in use for different purpose in the pharmaceutical industry, this property of scavenging radicals generated during radiolysis of water could be another application of such compounds [39, 40, 41, 42]; to provide protection to cells against radiation. The findings mentioned above for HPIA are important since experimental evidence suggests it is able to protect double stranded calf thymus DNA from radiation-induced damage. Hence, findings of this study may be used to develop molecules that can provide protection against radiation-induced damage.

It is also important to realize how a molecule like HPIA could be active as a radio-protective agent. To be a good radio-protector, a molecule should scavenge free radicals generated in solution when exposed to γ-radiation. This is possible either through direct scavenging of radicals formed in solution or by reversing a damage caused to a target. To establish the mechanism operative in case of HPIA that prevents radiation-induced damage of thymine and modification of calf thymus DNA, a study based on free radical reactions became necessary.

### 3.3 Mechanism of protection of radiation-induced DNA damage

As mentioned earlier, radiation-induced damage of DNA is a consequence of the radiolysis of water that generates ROS, most of which are free radicals. Scavenging them is important as they are harmful. It is therefore important to either remove them as soon as they are formed or render them inactive so that they cannot interact with possible targets. Since HPIA provides protection to DNA from radiation-induced damage, it was necessary to study different aspects pertaining to radical scavenging and suggest a mechanism by which HPIA prevents radiation induced damage. Radical scavenging activity of HPIA was examined by techniques like DPPH scavenging assay that was followed by UV-Vis spectroscopy, EPR spectroscopy and DCF-DA assay performed on WI 38 lung fibroblast cells.

#### 3.3.1 Assessment of scavenging of DPPH by UV-Vis spectroscopy

**Figure 6:**
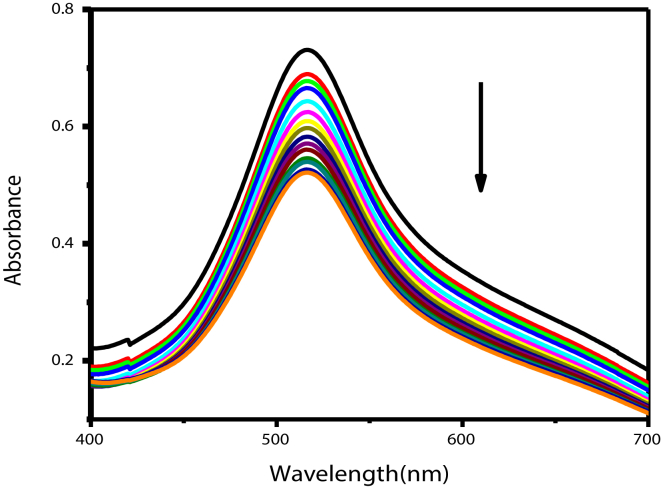
Changes in the UV-vis spectrum of DPPH (60 μM) in presence of HPIA (0–30μM) in ethanol.

Free radical scavenging of HPIA was investigated by allowing it to interact with DPPH, a stable free radical making use of electronic spectroscopy [34, 35, 36]. Radical scavenging was followed at 517 nm for different concentrations of HPIA. The ability of HPIA to scavenge DPPH in solution was realized from Figure 6. The deep violet color of DPPH gradually disappeared and a pale yellow color developed in solution as free radicals got neutralized providing a visual monitoring of the reaction. Concentration of the DPPH radical present in solution could therefore be realized from the change in absorption at 517 nm.

In the second pathway, in a subsequent step two distinctly different entities would exist in solution following the quenching of DPPH•. Whatever the mechanism, the conclusion is that HPIA has the ability to quench free radicals in solution which was realized using DPPH•.

#### 3.3.2 Assessment of scavenging of DPPH by EPR spectroscopy

**Figure 7:**
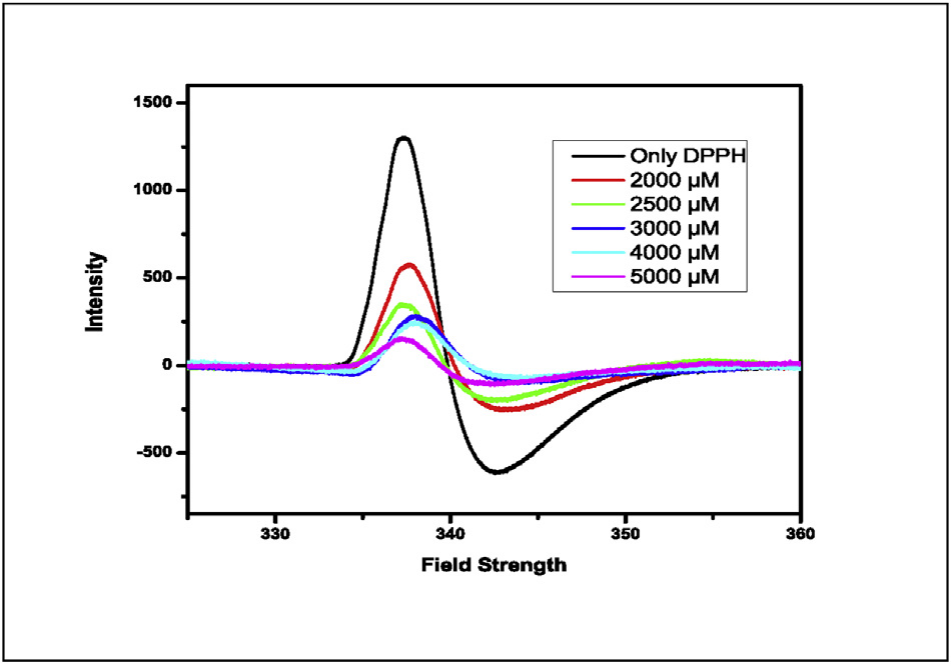
EPR spectra of DPPH (6 mM) with different concentrations of HPIA (0–5mM) in ethanol.

The EPR signal of DPPH was used to monitor the free radical scavenging activity of HPIA [21, 22, 23, 42, 43, 44]. Figure 7, shows for the same initial concentration of DPPH, increasing concentrations of HPIA lead to gradual decrease in its EPR response suggesting that with an increase in concentration of HPIA, a substantial amount of DPPH radical was no more present. Conversion of DPPH• to reduced DPPH might occur in two ways. One through adduct formation whereby one N atom of the azo bond in HPIA forms a bond with the nitrogen atom of DPPH• or with either of the benzene rings of DPPH• at the para position containing a lone electron [36]. Such adduct formation immediately destroys the radical character of DPPH•. The new compound formed from HPIA would have a lone electron localized on the other nitrogen atom of the azo bond. The other path for quenching DPPH• could be through the transfer of an electron from a nitrogen atom of the azo bond in HPIA to DPPH•. As a consequence the nitrogen atom on which the lone electron was present in DPPH• becomes N¯ in reduced DPPH converting HPIA to a radical-cation (HPIA·^+^).

#### 3.3.3 Measurement of ROS by the DCF-DA assay

Reactive oxygen species (ROS) are also responsible for damage caused to DNA within cells and these are generated in good amount following irradiation. Presence of ROS was estimated in WI-38 lung fibroblast cells with the help of the DCF-DA assay that measures fluorescence due to DCF [24, 25, 26, 27]. Cells were previously treated with HPIA using three different concentrations (1 μM, 5 μM, and 10 μM) for 30 min. The experiment was performed under two separate heads i) when ROS profile was estimated based only on ROS present in WI-38 lung fibroblast cells and ii) when ROS was estimated after it was irradiated with ^60^Co γ rays with a dose of 2 Gy. A dose of 2 Gy was selected to make the study relevant to biological systems with particular reference to aspects like radiotherapy [45, 46]. The ROS profile in each case was done for two different times, one immediately after treatment of cells referred to in the discussion as that performed at 0 h and another after 4 h.

**Figure 8:**
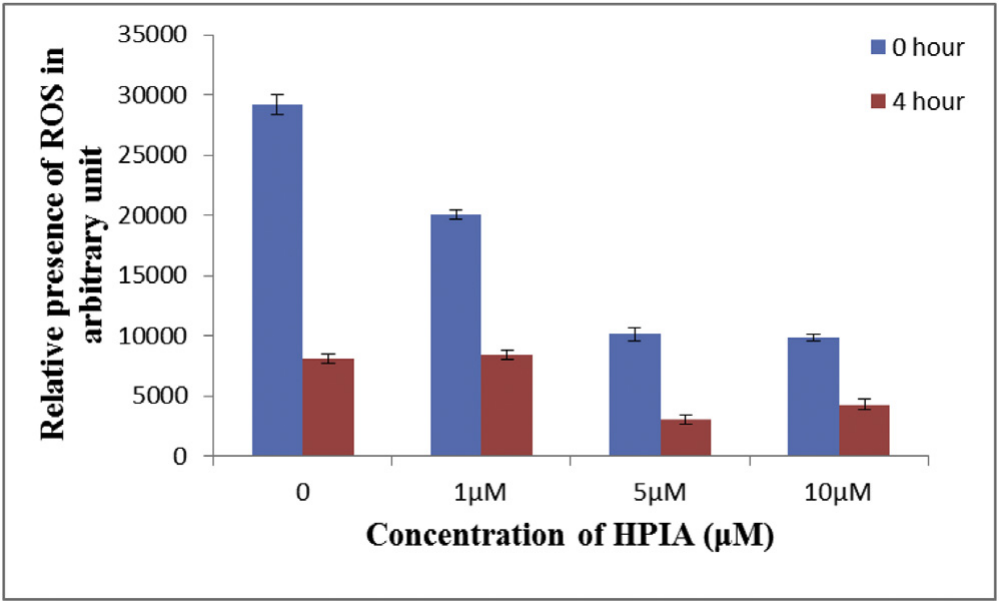
Effect of HPIA on ROS generation induced by ^60^Co γ rays on a WI-38 lung fibroblast cell line that was followed by the DCFDA assay using fluorescence spectroscopy.

When ROS present in WI-38 lung fibroblast cells were measured immediately i.e. 0 hour in the absence of radiation it was seen that for 1 μM HPIA, quenching was almost negligible while for the measurement made after 4 h it was higher. Results indicate 1 μM HPIA was not sufficient to quench the formation of extra ROS generated in the next four hours. However, with increase in concentration of HPIA to 5 μM and 10 μM respectively, values obtained for ROS decreased in a manner expected both for 0 h and 4 h indicating increased concentrations of HPIA (5 μM and 10 μM) were sufficient for ROS quenching. The experiment clearly showed the ROS quenching ability of HPIA (Figure 8).

ROS was induced in WI-38 lung fibroblast cells with the help of ^60^Co γ rays at a dose of 2 Gy and subsequently followed by the DCF-DA assay. Monitoring of fluorescence of DCF reveals when HPIA was not used, generation of ROS at 0 h far exceeds that at 4 h (Figure 8). This is because at 0 h i.e. immediately after irradiation there is a high concentration of ROS which after 4 h for irradiated cells decrease, as metabolic activity of irradiated cells decrease substantially. Moreover, no new ROS is generated by such cells themselves. However, the ROS quenching mechanism of cells remain active; rather they become more active to be able to decrease the increased presence of ROS (following irradiation) in such cells. Therefore, for irradiation provided in the absence of HPIA, the assay performed at 4 h indicates that the presence of ROS was significantly less. When irradiation was provided in presence of HPIA, decrease in ROS was much more systematic at all concentrations of HPIA used; values recorded at 4 h being significantly less than that at 0 h. The experiment showed that the ROS quenching ability of HPIA was significant at 5 μM and beyond it. Results obtained for cells irradiated in the presence of 1 μM HPIA was almost comparable to that when no compound was present during irradiation; the only difference being, amount of ROS present at 0 h was slightly less when HPIA was present which is clear from the above discussion.

## 4 Conclusion

Free radical scavenging and antioxidant activities of HPIA were examined. The study shows HPIA protects radiation-induced damage of thymine and prevents modification of double stranded calf thymus DNA compared to situations when experiments were performed on the same target in absence of HPIA. To investigate the mechanism of action of HPIA, DPPH radical quenching and DCF-DA assay were performed. Results show HPIA scavenged DPPH radical and depleted ROS generation in WI 38 lung fibroblast cells. Antioxidant efficacy of HPIA could be concluded from evaluation of radio-protection on thymine and calf thymus DNA following gamma irradiation of such targets in the absence and presence of HPIA. Results obtained are comparable to those of flavonoids. The DCF-DA assay further indicates HPIA mitigates reactive oxygen species−induced damage to cells by scavenging them. The DPPH scavenging ability of HPIA increased in a dose-dependent manner. A positive correlation of radio-protection with antioxidant activity due to HPIA was observed following different studies undertaken. At a concentration as low as 1 μM efficient radio-protection was observed due to HPIA on thymine and calf thymus DNA which is encouraging. However, the same concentration of 1 μM was not sufficient for ROS depletion for which slightly higher concentrations were required. Results suggest HPIA may be tried as an antioxidant supplement during radiotherapy for protection of normal cells.

## Declarations

### Author contribution statement

Durba Ganguly, Ramesh C Santra, Swagata Mazumdar: Performed the experiments; Analyzed and interpreted the data.

Abhijit Saha: Contributed reagents, materials, analysis tools or data.

Parimal Karmakar: Conceived and designed the experiments; Analyzed and interpreted the data.

Saurabh Das: Conceived and designed the experiments; Analyzed and interpreted the data; Wrote the paper.

### Funding statement

Saurabh Das was supported by 10.13039/501100010426UGC-DAE CSR, KC Collaborative Research Scheme (UGC-DAE-CSR-KC/CRS/13/RC03/0886RCS) Durba Ganguly was supported by a project fellowship from 10.13039/501100010426UGC-DAE CSR, KC Collaborative Research Scheme. Ramesh C Santra was supported by a Senior Research Fellowship from 10.13039/501100001501UGC. Saurabh Das was supported by the RUSA 2.0 program operating at 10.13039/100009589Jadavpur University (Ref. no. R-11/438/19 dated 30.05.2019) Saurabh Das was supported by 10.13039/501100001501UGC, New Delhi as part of UPE II, Jadavpur University and the UGC-CAS II program.

### Competing interest statement

The authors declare no conflict of interest.

### Additional information

No additional information is available for this paper.
